# Endoscopic management of colonic diverticulitis with fecalith impaction: a novel suction technique

**DOI:** 10.1055/a-2584-1946

**Published:** 2025-05-06

**Authors:** Shihe Hu, Jiyu Zhang, Deliang Li, Huige Wang, Dan Liu

**Affiliations:** 1 191599 Department of Gastroenterology and Hepatology, The First Affiliated Hospital of Zhengzhou University, Zhengzhou, China

A 40-year-old man presented to our hospital with a 1-year history of recurrent left lower abdominal pain. A computed tomography (CT) scan revealed multiple colonic diverticulitis ( Fig. 1 **a** , Video 1 ). Colonoscopy confirmed the presence of fecalith impaction within the diverticula. Initially, we attempted to extract the fecaliths using a short transparent cap for suction ( Fig. 1 **b** ). However, this approach proved challenging due to the narrow openings of the diverticula. Therefore, we switched to a long transparent cap, which allowed better visualization of the diverticulum openings and facilitated alignment of the fecaliths with the axis of the endoscope’s working channel for effective suction ( Fig. 1 **c** ). The long cap enhanced suction power and provided sufficient internal space for efficient fecalith removal. Complete extraction from all diverticula was achieved without complications ( Fig. 1 **d** ). Subsequently, endoscopic band ligation (EBL) was performed on the inverted colonic diverticulum ( Fig. 1 **e** ). The patient reported significant improvement in abdominal pain with no adverse reactions. Follow-up colonoscopy at 1 month demonstrated complete mucosal healing at the treatment site ( Fig. 1 **f** ).

**Fig. 1:**
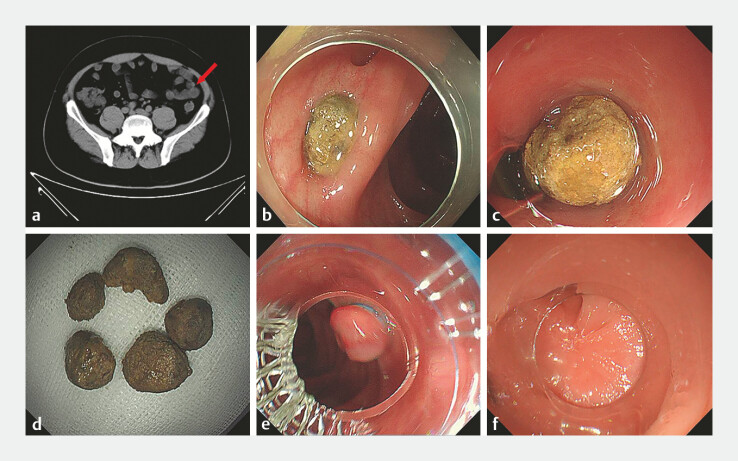
**a** CT scan revealed multiple colonic diverticulitis caused by calcified fecaliths. **b** Using a short transparent cap for suction. **c** Using a long transparent cap for suction. **d** The impacted fecaliths aspirated from diverticula. **e** The inverted diverticulum was exposed by suction with the transparent cap and ligated by EBL. **f** The colonic mucosa at the treatment site had healed completely. Abbreviation: EBL, endoscopic band ligation.

Endoscopic management of colonic diverticulitis with fecalith impaction: a novel suction technique.Video 1

Fecalith impaction is a leading cause of complicated colonic diverticulitis, often resulting in perforation or bleeding 1 . In cases where the diverticulum has a narrow opening, traditional methods such as biopsy forceps are often ineffective for fecalith removal. Based on our experience, the use of a long transparent cap significantly improves suction efficiency by exerting greater pressure and facilitating better expansion of the diverticulum opening. Furthermore, EBL is a simple, safe, and effective technique for the curative treatment of diverticula 2 3 . No abnormalities were observed during follow-up. In conclusion, long cap-assisted suction represents a promising and minimally invasive option for managing fecalith impaction in colonic diverticula, offering a safe and effective alternative to conventional techniques.

Endoscopy_UCTN_Code_TTT_1AQ_2AH

## Notes

Endoscopy E-Videos https://eref.thieme.de/e-videos
*E-Videos* is an open access online section of the journal *Endoscopy* , reporting on interesting cases and new techniques in gastroenterological endoscopy. All papers include a high-quality video and are published with a Creative Commons CC-BY license. Endoscopy E-Videos qualify for HINARI discounts and waivers and eligibility is automatically checked during the submission process. We grant 100% waivers to articles whose corresponding authors are based in Group A countries and 50% waivers to those who are based in Group B countries as classified by Research4Life (see: https://www.research4life.org/access/eligibility/ ). This section has its own submission website at https://mc.manuscriptcentral.com/e-videos .

## References

[1] Tursi A Scarpignato C Strate LL Colonic diverticular disease Nat Rev Dis Primers 2020 6 20 10.1038/s41572-020-0153-5 32218442 PMC7486966

[2] Kobayashi K Nagata N Furumoto Y Effectiveness and adverse events of endoscopic clipping versus band ligation for colonic diverticular hemorrhage: a large-scale multicenter cohort study Endoscopy 2022 54 735 744 10.1055/a-1705-0921 34820792 PMC9329063

[3] Li J Zhou Y Liu D Endoscopic band ligation: a simple, safe, and effective method for refractory diverticular bleeding (with video) Gastrointest Endosc 2025 101 220 221 39182522 10.1016/j.gie.2024.08.029

